# Hospital Mental Health Admissions in Women after Unsuccessful Infertility Treatment and *In Vitro* Fertilization: An Australian Population-Based Cohort Study

**DOI:** 10.1371/journal.pone.0120076

**Published:** 2015-03-25

**Authors:** Louise M. Stewart, C. D’Arcy J. Holman, James B. Semmens, David Preen, Qun Mai, Roger Hart

**Affiliations:** 1 Centre for Population Health Research, Faculty of Health Sciences, Curtin University, Bentley, Western Australia, Australia; 2 School of Population Health, The University of Western Australia, Crawley, Western Australia, Australia; 3 School of Women’s and Infants’ Health, The University of Western Australia, King Edward Memorial Hospital, Subiaco, Western Australia, Australia, and Fertility Specialists of Western Australia, Bethesda Hospital, Claremont, Western Australia, Australia; University of Kansas Medical Center, UNITED STATES

## Abstract

**Objective:**

To examine the association between in vitro fertilization (IVF) and later admission to hospital with a mental health diagnosis in women who remained childless after infertility treatment.

**Methods:**

This was a population-based cohort study using linked administrative hospital and registry data. The study population included all women commencing hospital treatment for infertility in Western Australia between the years 1982 and 2002 aged 20–44 years at treatment commencement who did not have a recorded birth by the end of follow-up (15 August 2010) and did not have a hospital mental health admission prior to the first infertility admission (n=6,567). Of these, 2,623 women had IVF and 3,944 did not. We used multivariate Cox regression modeling of mental health admissions and compared women undergoing IVF treatment with women having infertility treatment but not IVF.

**Results:**

Over an average of 17 years of follow-up, 411 women in the cohort were admitted to hospital with a mental health diagnosis; 93 who had IVF and 318 who did not. The unadjusted hazard ratio (HR) for a hospital mental health admission comparing women who had IVF with those receiving other infertility treatment was 0.50 (95% confidence interval [CI] 0.40–0.63). After adjustment for age, calendar year and socio-economic status the HR was 0.56 (95% CI 0.44–0.71).

**Conclusions:**

IVF treatment is associated with a reduced risk of hospital mental health admissions in women after unsuccessful infertility treatment. This may be explained by the healthy cohort effect.

## Introduction

A diagnosis of infertility in the absence of successful treatment, and thus the ultimate realisation for a woman that she will not be a mother to her own biological children is a potential cause of unresolved grief and psychiatric morbidity. [1–7]

Feelings of depression and anxiety after unsuccessful in vitro fertilization (IVF) can result from a combination of the effects of IVF treatment: its invasive nature, altered levels of circulating hormones experienced and the attending uncertainty, as well as the state of involuntary infertility in which women find themselves. A number of studies [5–8] have compared levels of anxiety and depression in women after successful and unsuccessful IVF treatment. IVF treatment was common to all participants, suggesting that the increased levels of depression and anxiety observed in women who remained childless was most likely due to their enduring infertility rather than its treatment.

In this study we were specifically interested in the association between IVF treatment and a mental health diagnosis separately from its association with IVF treatment outcome (successful or unsuccessful). Rather than comparing women who had unsuccessful IVF with women who had IVF and gave birth, we compared nulliparous women who had IVF with nulliparous women who had non-IVF infertility treatment. In this way we were able to focus on the effects of IVF treatment, separately from the outcome of the treatment. We considered two alternative possibilities. The first was that a woman who does not eventually conceive after embarking on IVF treatment may be significantly more ‘driven’ to attempt to conceive in comparison to a woman who ceases fertility treatment prior to commencing IVF treatment and hence may be more vulnerable to mental distress. The alternative possibility was that a woman who has endured the stress of IVF treatment and undertaken this as a last resort may feel she has done all she can to achieve her objective of having a child and this final resolution may allow her to move on and find satisfaction in other areas of her life.

The aim of this study was to examine the association between IVF treatment and later admission to hospital with a mental health diagnosis in women who remained nulliparous after infertility treatment.

The outcome measure was a clinically diagnosed mental health condition of a severity that warranted admission to hospital. We did not aim to capture all cases of depression and anxiety but only those mental health conditions on the severe end of the spectrum that had not been successfully treated in primary or specialist outpatient care.

## Materials and Methods

### The Study Population

This was a population-based cohort study. The study population was a subset of a source population that included all women known to be resident in Western Australia (WA) who had a hospital admission for infertility investigation or treatment with a diagnosis of either infertility or procreative management (International Classification of Diseases (ICD)-9-CM 628.0–628.9; ICD-10-AM N97.0-N97.9 or ICD-9-CM V26.1-V26.9; ICD-10-AM Z31.1-Z31.9) in the years 1982–2002 when they were aged between 20 and 44 years. All women in the study population had a hospital admission with a diagnosis of either infertility or procreative management. Some of the women in the study population also had IVF treatment while others did not. This population has been previously described. [9–12] The study population included only women in the source population who did not have a recorded birth before the end of follow-up and excluded women who had a hospital mental health admission before their first infertility admission.

### Data Sources

Data for this study were sourced from the WA Data Linkage System which connects routinely collected data for the entire state using probabilistic matching and clerical review. The data linkage system is dynamic: links are updated as new information comes to light and the number of data sets available to link is steadily increasing. Data are made available for research in de-identified format. [13,14] This study made use of data from five different data collections. The Hospital Morbidity Data System (HMDS) provided data on all hospital admissions between 1980 and 2010 and diagnoses contained in this were used to identify the source population, the outcome variable (a mental health admission) and covariates including age and socio-economic status. IVF treatment was identified from the HMDS using diagnostic codes V26.1, V26.8 (ICD-9CM) and procedure codes 81.64 (ICD-8), 65.91, 65.99, 66.99, 69.99, and 70.12 (ICD-9CM) for the period 1982–1992, and from the Reproductive Technology Register for the period 1993–2002. Deaths were identified from the WA Deaths Registrations (1982–2010) and births from the Midwives Notifications (1980–2010). The WA Electoral Roll was used to identify and exclude women who were known to have moved out of the State (1988–2010) and were hence lost to follow-up. Women with an out-of state address on their hospital records were also excluded.

Socio-economic status was derived from the address recorded on the woman’s hospital record at the first infertility admission. The Index of Economic Resources was selected to represent socio-economic status in this study. [15]

### The Outcome Variable

The outcome of interest was a hospital admission where the principal diagnosis (i.e. the main reason for admission to hospital) was a mental health diagnosis. Diagnoses were coded according to ICD versions 9 and 10. ICD-9-CM coding applied to all records up to 30 June 1999 and ICD-10-AM coding from 1 July 1999. A mental health diagnosis included any condition coded within the range 290–319 (ICD-9-CM) and F00-F99 (ICD-10-AM). Women could be hospitalised on a number of occasions with a mental health diagnosis. In this analysis, we considered only the first mental health admission with follow-up censored at the date of that event.

### Exposure Variable and Potential Confounders

The exposure variable was IVF treatment, including both in vitro fertilization and intracytoplasmic sperm injection (ICSI). Covariates included age, calendar year and socio-economic status. Age, calendar year and socio-economic status at the start of follow-up were included in the regression model as categorical variables. Age was grouped into 5-year age groups and calendar year into 3-year groupings. We compared women in the upper quartile of the Index of Economic Resources with women in the lower three quartiles combined. [15]

### Data Analysis

Data were analysed, and hazard ratios (HRs) and their corresponding 95% confidence intervals (CIs) were estimated using Cox regression analysis. IVF treatment could occur at the start of follow-up (i.e. at the first infertility admission) or sometime later. Because of this, IVF was included in the regression model as a time dependent variable. Univariate and multivariate analyses were performed.

Women who did not have a recorded birth were followed from their first infertility admission to their first hospital mental health admission, death, or the censor date of 15 August 2010, whichever came first. All analyses were performed using SPSS version 21. Data were analysed anonymously.

### Ethics Statement

This study received ethics approval from The University of Western Australia Human Research Ethics Committee (Ref: RA/4/1/1515), and the Department of Health WA Human Research Ethics Committee (Reference no: 2012/29). The project received approval from the Reproductive Technology Council (WA).

## Results

### The Study Population

The initial study population, comprising a total of 7,149 women, included all women seeking hospital infertility treatment who did not have a recorded birth up to the end of follow-up. We excluded 582 women who had a hospital admission for a mental health condition prior to their first infertility admission, as they were no longer at risk of a first mental health admission after infertility treatment, leaving a total of 6,567 women. Among the excluded women, 25% went on to have IVF and 18% were in the upper quartile of the Index of Economic Resources. In comparison, of those included in the study population, 40% had IVF (the remainder had infertility investigation and treatment but not IVF) and 24% were in the upper quartile of the Index of Economic Resources (Table 1). The proportion of women having IVF increased over the duration of the study (from 1982–2002): in 1982, 35% of women who commenced treatment for infertility had IVF compared with 43% commencing in 2001. Women were followed from their first infertility admission when they were on average 33 years of age to an average age of 50 years (Table 1). During this time, a total of 411 women were admitted to hospital for treatment of a mental health condition.

**Table 1:** Characteristics of the study population.*

Characteristic	All women seeking fertility treatment	Women not undergoing IVF	Women undergoing IVF
Number of women	6,567	3,944 (60%)	2,623 (40%)
Number with a mental health admission (%)	411 (6.3%)	318 (8.1%)	93 (3.5%)
Number of women in the highest quartile of the Index of Economic Resources (%)	1,557 (24%)	815 (21%)	742 (28%)
Mean duration of follow-up (years) †	17.3 ± 6.6	17.0 ± 6.8	17.9 ± 6.3
Total duration of follow-up (person-years)	113,872	66,983	46,889
Mean age at first infertility admission (years)	32.9 ± 5.5	32.5 ± 5.7	33.5 ± 5.0
Mean age at first mental health admission (years)	38.0 ± 8.6	37.1 ± 8.6	40.9 ± 7.9
Mean age at end of follow-up (years)	50.1 ± 8.2	49.4 ± 8.6	51.2 ± 7.3

* The study population includes all women commencing hospital investigation and treatment for infertility between 1982 and 2002 when they were 20–44 years of age who did not have a recorded birth at the end of follow-up and did not have a hospital mental health admission before their first infertility admission. Information on exposures and outcomes was collected over a period of 30 years, from 1980 to 2010.

† All means reported ± SD.

A mental health admission included any diagnosis within the range 290–319 (ICD-9) and F00-F99 (ICD-10); however there were very few admissions for most of these diagnostic codes, with a concentration around specific diagnoses. Table 2 summarises the mental health diagnoses recorded in this population. These were mostly related to depression, anxiety, adjustment disorders, reaction to severe stress and disorders due to drug and alcohol use (Table 2). These categories made up 84% of the total number of principal diagnoses recorded at the first mental health hospital admission.

**Table 2:** Mental health diagnoses in women who remained nulliparous after infertility treatment and IVF.

ICD-9 and ICD-10 codes *	Description	Number (% of total) ^†^
300, 308–309, F41, F43	Anxiety disorders, adjustment disorders and reaction to severe stress	154 (37%)
296.2–296.3, 311.0, 311.9, F32–F34	Depressive disorders	123 (30%)
291–292, 303–305, F10–F19	Mental and behavioural disorders due to drug or alcohol use	68 (17%)
295.4, 295.6, 296.0, 296.1, 296.4, 296.5, 297.8, 298, F20–F31	Schizophrenia, bipolar disorders and psychosis	40 (10%)
293.8, 301, 306–307, F03, F05, F06, F40, F44, F45	Other unrelated categories	26 (6%)

* ICD-9 codes apply to admissions up to 30 June 1999 and ICD-10 codes apply to admissions thereafter.

† It was not possible to report numbers of admissions separately for women who did and did not have IVF due to confidentiality requirements as totals were less than 10 in some categories.

### Cox Regression Analysis

In univariate (unadjusted) analysis, we found that women who had IVF treatment had a rate of hospital mental health admission that was half that of women who were treated for infertility but did not have IVF (HR = 0. 50; 95% CI 0.40–0.63).

In adjusted analysis we found that women who had IVF treatment had 0.56 times the rate of mental health admissions of women who had non-IVF infertility treatment (HR = 0.56; 95% CI 0.44–0.71) (Table 3).

**Table 3:** Association between IVF treatment and mental health hospital admissions in women who remained nulliparous after infertility treatment.

Variable	Hazard Ratio (95% confidence interval) *
**IVF**	
IVF (no)	1.00 (reference)
IVF (yes)	0.56 (0.44–0.71)
**Socio-economic status**	
Low socio-economic status †	1.00 (reference)
High socio-economic status	0.73 (0.56–0.96)

* Hazard ratios estimated from a Cox regression model that includes the variables listed plus age group and calendar year group (both included as categorical variables).

† Women in the lower three quartiles of the Index of Economic Resources were categorised as being of low socio-economic status; women in the upper quartile of the Index of Economic Resources were categorised as being of high socio-economic status.

High socio-economic status was associated with a reduced rate of mental health admissions (HR = 0.73; 95%CI 0.56–0.96) (Table 3).

We included age and calendar year at the start of follow-up in the multivariate regression model as categorical variables. Neither variable followed a strict linear trend but there was a general trend towards a decrease in risk of a mental health related hospital admission with increasing age and later calendar year.

## Discussion

In this study we found that among women who remained childless after infertility treatment, those who had IVF treatment were around half as likely to be admitted to hospital for a mental health condition as women who had infertility treatment but not IVF. This association remained even after controlling for other factors including age, year of commencing infertility treatment and socio-economic status.

We hypothesise that these findings can be explained by the “healthy cohort effect”. This concept is often encountered in occupational epidemiology where it is termed the “healthy worker effect” [16]—people who are employed are, as a group, healthier than the general population because employers are likely to select healthy employees and people must remain healthy to continue to be employed—those who become ill may be forced to leave their place of work. The healthy cohort effect has also been invoked to account for the apparent cardio-protective effect of hormone replacement therapy (HRT) recorded in observational studies—women who choose HRT also adopt other healthy behaviours and it is likely that it is this, rather than HRT treatment, that accounts for their reduced risk of cardiovascular disease. [17]

There are a number of ways in which the healthy cohort effect could explain the findings of a reduced rate of hospital mental health admissions in women who had IVF. We know that IVF is emotionally and physically demanding. Therefore, it is possible that only mentally robust women select IVF treatment. Women who choose to undergo IVF may be more mentally resilient at the start of treatment than women who do not, and this is reflected in their later reduced risk of a hospital mental health admission. However, we made one observation that would suggest that this argument cannot explain the entire effect. In this study we excluded women who had a mental health admission prior to their first infertility admission. Of the excluded women, 25% went on to have IVF. This proportion is smaller than the 40% we observed in the study cohort, but it does show that needing hospital treatment for mental health problems is not a barrier to commencing IVF.

A second explanation is that women who undergo IVF are more proactive in seeking care when they need it. Therefore, if they suffer mental health problems after infertility treatment they would be more likely to seek assistance from health care professionals and consequently not get to the point where they would require admission to hospital. It is this behaviour that explains their reduced risk of a hospital mental health admission. In Australia, women with mental health problems would usually first consult a General Practitioner (GP). The GP would offer counselling and/or treatment and if necessary, a referral to a psychiatrist or psychologist. [18] Admission to hospital would require a referral from a treating physician or via the hospital emergency department. In this context, mental health problems can be viewed as “ambulatory care sensitive conditions” [19] i.e. conditions that could be treated effectively in primary health care so that subsequent hospitalisation is avoided. In support of this hypothesis, we observed that women of higher socio-economic status were less likely to be admitted to hospital with mental health problems. The association between socio-economic status and health has been repeatedly demonstrated. [20–22] We suggest that socially advantaged women in our study have the financial and personal resources to seek out appropriate treatment in primary care and thereby avoid hospital treatment and further, we suggest that that this health seeking behaviour is even more pronounced in women who have IVF. Even after the effects of socio-economic status were taken into account, women in our study who had IVF were only about half as likely to be admitted to hospital with a mental health diagnosis as women who had non-IVF infertility treatment.

Other authors have also found evidence for a healthy cohort effect. Two studies that compared women undergoing IVF treatment with controls drawn from the general population, one based in Finland [23] and the other in Denmark [24] found that women undergoing IVF were less likely to be admitted to hospital with a mental health condition than controls matched for age [24], or age, socioeconomic status and marital status. [23] Both authors suggested that their findings could be partly explained by the healthy cohort effect. In another Finnish study, Klemetti [25] found that all-cause and cardiovascular specific mortality in women undergoing IVF or ovulation induction was lower than in the general female population while in an Australian study, Venn et al [26] identified a lower than expected mortality rate among women undergoing IVF. Both proposed the healthy cohort effect as a plausible explanation for their findings. Kristiansson et al [27] compared parous women who had IVF with parous women who did not and found a reduced risk of cervical cancer in the women who had IVF, a finding which may also be partly explained by the healthy cohort effect.

Previous studies have found that women who remain childless after IVF appear to be at increased risk of emotional distress, depression and anxiety [1,2,4–7] although a number of studies have reported declining levels of anxiety and depression over time. [1,6,8] These studies have measured how women feel; the present study measured how women coped. Combining the findings from these previous studies with the present study, it appears that women who remain childless after IVF may experience more depression and anxiety than women whose IVF treatment is successful. However, among women who remain childless, those who have IVF are less likely to be admitted to hospital with a mental health condition than women who have non-IVF infertility treatment. It may be that women who undergo IVF find it a stressful process, experiencing symptoms of depression and anxiety as a result, but they have the health seeking behaviour that enables them to get treatment if and when they need it before serious problems requiring hospitalisation can develop.

This study has a number of strengths and limitations. Limitations include the fact that this was a non-experimental study. In common with all observational studies, it is possible that underlying risk factors may be unevenly distributed between the two groups under study. If these risk factors are also related to the outcome (a mental disorder requiring admission to hospital) then confounding could have occurred and the estimates would be biased. We attempted to minimise the potential for such confounding, and this is a strength of the study design, by making comparisons within a cohort of women undergoing infertility treatment rather than comparing women who had IVF with the general population. Potential confounders such as parity, history of infertility and reason for infertility were more likely to be evenly distributed between the two groups under comparison than between women undergoing IVF and the general population. This is because the general population is made up of parous and nulliparous women and fertile and infertile women whereas our cohort of infertile women comprised only nulliparous women with a diagnosis of infertility. Nevertheless, it is likely that women who have IVF differ from women who have non-IVF infertility treatment. Tubal factor and male factor infertility may require IVF treatment whereas women with ovulatory disorder and couples where the male partner is azoospermatic may require ovulation induction with insemination treatment and donor insemination treatment respectively. Even so, confounding can only occur if these factors are also related to the outcome (a mental disorder). The cost of treatment and accordingly the ability to pay may also differ between couples who choose IVF and those who do not; however, we included a measure of socio-economic status in our analysis in order to adjust for this. Inclusion of socio-economic status is a further strength of this study. Socio-economic status is a powerful and pervasive health-related risk factor [21] yet it is rarely included in analyses such as the present one. Importantly, we did not simply include socio-economic status as a confounder; we also discussed the implications of our findings with respect to socio-economic status.

There was some potential for misclassification in this study. The earliest year of entry into the study was 1982. We had data on births from 1980. Births to women in the cohort prior to 1980 were unknown to us and women who gave birth before 1980 may have been incorrectly included in the study population. Secondary infertility was more common among women who did not have IVF. Therefore we would have been more likely to include parous women in the non-IVF group than the IVF group. For mental health diagnoses associated with nulliparity our hazard ratio estimates would therefore have underestimated the reduced rate among women having IVF. Similarly, women who had a mental health diagnosis prior to 1980 would have been incorrectly included in the study; however numbers of these would have been small as we observed very few mental health admissions in 1982–1983. In addition, we did not have any information on adoption. Nulliparous women in our study were only those who did not give birth to a child. It is likely that some of the women in our study were mothers to adopted children. However, adoption rates in Australia are low, with numbers continuing to decline: in recent years, only around 40 children were adopted each year in Western Australia. [28] There may have been some misclassification of exposure and loss to follow-up in women who moved into or out of WA between 1982 when the study commenced and 1988 when we were able to identify movement into or out of the state through electoral roll records. However, we also used the address recorded on hospital records to exclude women with an out-of-WA address and therefore we expect this level of misclassification to be small. Some women may have had non-IVF treatment between 1982 and 2002 and later had IVF. This later IVF treatment would be unknown to us and these women would have been misclassified. However, most women had IVF treatment soon after the initial infertility admission. We therefore expect that the number of women misclassified in this way would be small.

A further limitation of this study is that it did not identify all cases of depression and anxiety in the population, only those that required admission to hospital. However, the aim of this study was not to identify all cases of depression and anxiety but rather to focus on those that were serious enough to require admission to hospital. Other strengths include the fact that the outcome variable was a clinical diagnosis of a mental health disorder and that the study population was large with long-term follow-up.

The results of this study show that among women who remain childless after infertility treatment, those who have IVF are less likely to require hospital treatment for a mental health disorder than those who have non-IVF infertility treatment. The findings of this study provide evidence for a healthy cohort effect.
